# Economic Burden of Multimorbidity and Polypharmacy Among Geriatric Patients: A Single-Center Experience From Odisha, India

**DOI:** 10.7759/cureus.74752

**Published:** 2024-11-29

**Authors:** Kulwant Lakra, Mamata Pandey, Satyabrata Meher, Bimal K Panda, Raj K Meher, Deepak Panigrahi, Ravindra Kumar Chowdury, Sujata Panda, Sanjay Kumar Mahapatra

**Affiliations:** 1 Community Medicine, Veer Surendra Sai Institute of Medical Sciences and Research, Burla, Sambalpur, IND; 2 Multidisciplinary Research Unit, Veer Surendra Sai Institute of Medical Sciences and Research, Burla, Sambalpur, IND; 3 Anaesthesiology, Veer Surendra Sai Institute of Medical Sciences and Research, Burla, Sambalpur, IND; 4 Surgery, Veer Surendra Sai Institute of Medical Sciences and Research, Burla, Sambalpur, IND; 5 General Medicine, Veer Surendra Sai Institute of Medical Sciences and Research, Burla, Sambalpur, IND; 6 Ophthalmology, Veer Surendra Sai Institute of Medical Sciences and Research, Burla, Sambalpur, IND; 7 Ear, Nose, and Throat, Veer Surendra Sai Institute of Medical Sciences and Research, Burla, Sambalpur, IND; 8 Urology, Veer Surendra Sai Institute of Medical Sciences and Research, Burla, Sambalpur, IND

**Keywords:** chronic kidney disease (ckd), diabetes, economic burden, geriatric patients, hypertension, multimorbidity, polypharmacy

## Abstract

Background

Multimorbidity and polypharmacy are common in older adults and pose a considerable challenge to the health and social care system. They are complex and interrelated concepts in geriatric care that require early detection and patient-centered shared decision-making by multidisciplinary, team-led comprehensive geriatric assessment (CGA) across all health and social care settings. The primary objective of this study is to investigate the economic burden of multimorbidity and polypharmacy among geriatric patients.

Methodology

A cross-sectional study was conducted from July 2021 to June 2022 involving a total of 470 male and female respondents aged more than 60 years. Five different departments of Veer Surendra Sai Institute of Medical Sciences and Research (VIMSAR), Burla were selected for the study. Data were collected by interviewing and clinically examining 470 randomly selected geriatric patients who came to different outpatient departments of VIMSAR, Burla. Statistical analysis was performed with SPSS software version 21.0 (IBM Corp., Armonk, NY, USA). P-values <0.05 were considered statistically significant.

Results

Of the 470 study participants, farming (126, 26.8%)and other (149, 31.7%) occupations were the maximum. Overall, 97 (20.6%) respondents belonged to the upper socioeconomic class, 148 (31.5%) to the upper middle class, 131 (27.9%) to the lower middle class, and 94 (20%) to the lower socioeconomic class. A single chronic disease was higher in respondents from the upper class at 87 (89.69%) and upper middle class at 128 (86.48%). Multimorbidity diseases were higher in the lower middle (28, 21.37%) and lower (24, 25.53%) income group patients. Diabetes was more common in males at 110 (30.81%) in comparison to females at 7 (6.19%). Hypertension was also higher in males at 90 (25.21%) compared to females at 21 (2.65%). Chronic kidney disease was seen in 41 (28.01%) males and 13 (11.50%) females. People with a smokeless tobacco habit (165, 35.11%) and a family history of diabetes (99, 21.06%) and hypertension (117, 24.89%) were suffering from single chronic diseases.

Conclusions

Multimorbidity has a high incidence in old age. It is associated with substantially higher healthcare utilization and social care costs among older adults in Odisha. With the incidence of multimorbidity rising, especially as the population ages, we require healthcare systems that are developing to deal with the rising challenges related to multimorbidity and the relevant healthcare and societal costs.

## Introduction

Multimorbidity, defined as the coexistence of multiple long-term diseases in the same individual, has increased rapidly. Chronic conditions are closely related to aging; however, only select aspects of multimorbidity have been investigated, such as the prevalence and consequences of coexisting diseases [1]. Around 55-98% of the geriatric population is affected by multimorbidity [2,3]. It has been recognized that there is an apparent relationship between rising numbers of chronic diseases and disability [4-6], poor quality of life [6,7], and elevated healthcare management [8,9]. Multimorbidity, defined as the coexistence of two or more chronic medical conditions in an individual, is a growing public health concern for healthcare systems worldwide [10]. Complexity in understanding multimorbidity arises from the fact that diseases can coexist in the same individual for several reasons, including random chance, common risk factors, or iatrogenic complexities [11]. Indeed, aging is the strongest risk factor for many chronic diseases. Current healthcare systems are largely designed to treat a single disease and use multiple services to manage multimorbidity. Poor coordination and integration in medical care cause a lack of continuity in treatment, and disorders not designated as the primary condition are often undertreated.

In contrast to comorbidity, multimorbidity refers to the simultaneous existence of two or more chronic health conditions that are independent of a primary or underlying illness. To better match medical treatment with the requirements of people with various health disorders, a comprehensive study of the epidemiology of multimorbidity in the general population is essential. Research indicates that multimorbidity may occur across all age demographics, with special emphasis on the older population owing to its elevated occurrence in this group [12].

Objectives

The objective of this investigation was to identify multimorbidity tendencies in both older patients and other demographic and socioeconomic categories. Furthermore, we examined the effects of multimorbidity using diverse common statistical techniques. Demographic data, including date of birth, gender, geographic location, socioeconomic position, baseline vital signs (e.g., smoking and alcohol use), and medical history (e.g., medical conditions and diagnosis dates), were extracted from patient records.

## Materials and methods

Place of study

This was a cross-sectional, descriptive, epidemiological, community-based study. The study was approved by the Institutional Ethics Committee, Veer Surendra Sai Institute of Medical Sciences and Research (VIMSAR), Burla (approval number: 19283/20.02.20 IFO-273/19). Data collection was done on the outpatient department (OPD) days of a week over one year from July 2021 to June 2022 in different departments of VIMSAR, Burla (Community Medicine, General Medicine, Ophthalmology, ENT, and Urology). A systematic random sampling technique was used in this study. Individuals aged over 60 years who were conversant and comprehensible were included in the study.

Inclusion and exclusion criteria

All geriatric patients (ages >60 years) coming to different OPDs of VIMSAR, Burla were considered for inclusion in the study. To avoid duplication, each patient was given a unique identification number. For patients too ill to participate, or having debilitating physical or mental disabilities, data were collected from their attendants. Pregnant women and those who had already been interviewed previously under the present study were excluded from this study.

Sample size and sampling

Sample size (n) = (Z1 − α/2)2(p)(q)/d^2^, where n is the desired sample size; Z1−α/2 is the critical value, and a standard value for the corresponding level of confidence (at 95% confidence interval (CI) or 5% level of significance (type-I error), it was 1.96); P is the expected prevalence or based on previous research; q is 1 − p, and d is the margin of error or precision.

Assuming a prevalence of multimorbidity of 40% in the geriatric age group [13], a precision of 5%, and a no-response rate of 22%, the sample size was estimated to be 468, which was rounded to 470. The sample size was divided proportionately to individual OPD attendance, with 50 patients from Community Medicine, Ophthalmology, and ENT OPDs each, and 160 patients from General Medicine and Urology ODPs each.

Data collection

Data were collected by trained field investigators utilizing a pretested, predesigned, semi-structured questionnaire that served as the primary tool for data collection. The questionnaire was developed using Open Data Kit (ODK) software on Android tablets. The Multimorbidity Assessment Questionnaire for Primary Care (MAQ-PC) [14] tool was utilized to assess multimorbidity status, having been previously validated in the study population. Sociodemographic data encompassed gender (male and female), age groups (60-75, 76-85, >85 years), marital status (married, never married, divorced, widowed), religion (Hindu, Christian, Muslim, others), and education levels (no formal education, less than primary, primary school completed, secondary school completed, high school completed, college completed, post-graduation).

The respondents’ occupations (agricultural labor, farming, government employee, non-government employee, unemployed, self-employed), current work status (retired, full-time working, part-time working, homemaker), and socioeconomic status based on per-capita monthly income using the Kupuswami scale divided as upper, upper-middle, lower-middle and low were also collected. Additionally, data on frailty, healthcare utilization, hospitalization, and social security were collected following the standard census definitions of India. Data regarding personal habits, including smoking, smokeless tobacco use, and alcohol consumption, were gathered.

History of a prior diagnosis of arthritis/joint pain, hypertension, diabetes mellitus, heart diseases, chronic lung diseases, acid peptic diseases, chronic backache, stroke, blindness, deafness, dementia, alcohol disorder, cancer, chronic kidney diseases (CKDs), epilepsy, thyroid diseases, tuberculosis, and filariasis was recorded based on earlier diagnoses by a physician.

Data analysis

Recorded data were entered, checked, and analyzed using SPSS version 21.0 (IBM Corp., Armonk, NY, USA). Qualitative data were expressed as frequency and percentage. The chi-square test was utilized to analyze between two variables. P-values <0.05 were considered significant.

## Results

A total of 470 participants were included in this study (Table 1). Of the study participants, 357 (76.0%) were male, while 113 (24.0%) were female. The mean age of the respondents was 67.99 years (SD = 7.30), with ages ranging from 60 to 95 years. Regarding family structure, 376 (80.0%) belonged to joint families, 82 (17.4%) were from nuclear families, and 12 (2.6%) identified as single. Educational attainment varied among participants, with 149 (31.7%) being illiterate, 93 (19.8%) having less than primary education, 71 (15.1%) having completed primary school, another 71 (15.1%) having finished high school, 53 (11.3%) having completed secondary school, 30 (6.4%) having attended college, and only 3 (0.6%) having achieved postgraduate education. The majority of participants, 461 (98.1%), were Hindus. Occupationally, 126 (26.8%) respondents were engaged in farming. Regarding economic status, 148 (31.5%) respondents belonged to the upper middle class. For drinking water, the majority, 216 (46.0%), used hand pump water.

**Table 1:** Sociodemographic and clinical variables of study participants.

Characteristic		Number (n = 470)	Percentage
Age (years)	60–75	405	86.2
	76–85	54	11.5
	>85	11	2.3
Gender	Male	357	76.0
	Female	113	24.0
Family type	Joint	376	80.0
	Nuclear	82	17.4
	Single	12	2.6
Religion	Hindu	461	98.1
	Christian	2	0.4
	Muslim	6	1.3
	Other	1	0.2
Education	No formal education	149	31.7
	Less than primary	93	19.8
	Primary school	71	15.11
	Secondary school	53	11.3
	High school	71	15.1
	College	30	6.4
	Postgraduation	3	0.6
Occupation	Agriculture	12	2.6
	Farming	126	26.8
	Governmental employee	50	10.6
	Non-governmental employee	13	2.8
	Not working for pay	72	15.3
	Others	149	31.7
	Self-employed	48	10.2
Socioeconomic status	Upper	97	20.6
	Upper middle	148	31.5
	Lower middle	131	27.9
	Low	94	20
Main source of drinking water	Hand pump	216	46.0
	Public tap	22	4.7
	Tap	204	43.4
	Well	28	6.0
Multimorbidity	Yes	82	17.5
	No	388	82.6

Morbidity patterns

The clinical variables of the multimorbidity cluster analysis are shown in Table 2. Overall, 342 (84.44%) participants in the age group of 60-75 years had single chronic diseases, and 63 (15.55%) suffered from multimorbidity diseases. Of those in the age group of 76-85 years, single chronic diseases were noted in 45 (83.33%), and nine (16.66%) were suffering from multimorbidity diseases. However, of those in the age group >85 years, 1 (9.09%) had multimorbidity disease and 10 (90.91%) had a single chronic disease, with a significant difference (χ^2^ = 42.245 and p < 0.0001). When comparing male and female participants, males were significantly higher (303, 84.87%) and affected with single chronic diseases than females (85, 75.22%). However, in the case of multimorbidity diseases, females suffered higher (28, 24.78% ) than males (54, 15.13%), and it showed a significant difference (χ^2^ = 4.903 and p = 0.026). Regarding the family type, the nuclear family was affected more with a single chronic disease at 76 (92.68%) than joint family and single family, but the multimorbidity diseases were higher at 5 (41.67%) in the case of a single family than the joint and nuclear family, with a significant difference (χ^2^= 11.268 and p = 0.0036). The scheduled tribe (ST) participants suffered from single chronic diseases (12, 85.71%) than other religions, but the scheduled caste (SC) participants suffered higher in multimorbidity diseases (31, 20.81%) than other groups, with no significant difference (χ^2^ = 2.087, p= 0.0554). Single chronic diseases were higher in the illiterate study sample at 124 (83.22%). Multimorbidity was higher in less than primary educated participants at 25 (26.88%), with no significant difference among these groups (χ^2^ = 9.273 and p = 0.158). The agricultural labor respondents suffered higher with single diseases (11, 91.67%) than other occupations, with not working for pay (15, 20.83%) and self-employed (10, 20.83%) individuals suffering more with multimorbidity diseases than other occupations, with no significant difference (χ^2^ = 2.711 and p = 0.844). Regarding socioeconomic status, upper-class participants suffered more from single chronic diseases (87, 89.69%). On the other hand, lower-income participants suffered more from multimorbidity diseases (24, 25.53%), which showed a significant difference (χ^2^ = 10.690 and p = 0.0135).

**Table 2:** Clinical variables of the multimorbidity clusters. *: Statistically significant at p < 0.05.

Characteristics		Number of cases, N	Morbidity status, n (%)		Chi-square value (p-value)
			Single chronic disease, n (%)	Multimorbidity, n (%)	
Age	60–75years	405	342 (84.44%)	63 (15.56%)	42.245 (<0.0001)*
	76–85 years	54	45 (83.33%)	9 (16.67%)	
	>85 years	11	1 (9.09%)	10 (90.91%)	
Gender	Male	357	303 (84.87%)	54 (15.13%)	4.903 (0.026)*
	Female	113	85 (75.22%)	28(24.78%)	
Family type	Joint	376	305 (81.12%)	71 (18.88%)	11.268 (0.0036)*
	Nuclear	82	76 (92.68%)	06 (7.32%)	
	Single	12	07 (58.33%)	05 (41.67%)	
Ethnicity	General	83	68 (81.93%)	15 (18.07%)	2.087 (0.0554)
	OBC	224	190 (84.82%)	34 (15.18%)	
	SC	149	118 (79.19%)	31 (20.81%)	
	ST	14	12 (85.71%)	2 (14.29%)	
Education	No formal education	149	124 (83.22%)	25 (16.78%)	9.273 (0.158)
	Less than primary	93	68 (73.12%)	25 (26.88%)	
	Primary school	71	59 (83.10%)	12 (16.90%)	
	Secondary school	53	46 (86.79%)	7 (13.21%)	
	High school	71	62 (87.32%)	9 (12.68%)	
	College	30	27 (90%)	3 (10%)	
	Postgraduation	3	2 (66.67%)	1 (33.33%)	
Occupation	Agriculture	12	11 (91.67%)	1 (8.33%)	2.711 (0.844)
	Farming	126	105 (83.33%)	21 (16.67%)	
	Governmental employee	50	41 (82%)	9 (18%)	
	Non-governmental employee	13	12 (92.31%)	1 (7.69%)	
	Not working for pay	72	57 (79.17%)	15 (20.83%)	
	Others	149	124 (83.22%)	25 (16.78%)	
	Self-employed	48	38 (79.17%)	10 (20.83%)	
Socioeconomic status	Upper	97	87 (89.69%)	10 (10.31%)	10.69 (0.0135)*
	Upper middle	148	128 (86.48%)	20 (13.52%)	
	Lower middle	131	103 (78.63%)	28 (21.37%)	
	Low	94	70 (74.47%)	24 (25.53%)	

The 470 participants suffered from 28 different diseases, as shown in Table 3. Overall, 4 (0.85%) suffered from acid peptic diseases; 1 (0.21%) from alcohol disorder; 15 (5.95%) from arthritis; 1 (0.21%) from asthma and cough; 2 (0.42%) from blindness; 3 (0.63%) from cancer; 4 (0.84%) from chronic backache; 54 (11.48%) from CKD; 9 (1.91%) from chronic lungs diseases; 3 (0.63%) from dementia; 117 (24.89%) from diabetes; 22 (4.68%) from diabetes, hypertension, and weakness; 28 (5.96%) from ear problems and hypertension; 1 (0.21%) from epilepsy; 5 (1.06%) from eye problems; 1 (0.21%) from hernia and stomach pain; 8 (1.7%) from head reeling and weakness; 1 (0.21%) from headache; 4 (0.85%) from heart diseases; 111 (23.62%) from hypertension; 18 (3.82%) from hypertension and stomach problems; 42 (8.94%) from prostate concerns; 1 (0.21%) from skin problem and weakness; 3 (0.63%) from stomach pain; and 1 (0.63%) each from swelling over the nose, body pain, and weakness, thyroid, and tuberculosis each.

**Table 3:** Percentage of diseases among participants.

Type of diseases	Frequency, n (%)
Acid peptic diseases	4 (0.85)
Alcohol disorder	1 (0.21)
Arthritis	15 (3.19)
Asthma and cough	1 (0.21)
Blindness	2 (0.42)
Cancer	3 (0.63)
Chronic backache	4 (0.84)
Chronic kidney diseases	54 (11.34)
Chronic lungs diseases	9 (1.91)
Deafness	9 (1.89)
Dementia	3 (0.63)
Diabetes	117 (24.89)
Diabetes, hypertension, weakness	22 (4.68)
Ear problem, hypertension	28 (5.96)
Epilepsy	1 (0.21)
Eye problem	5 (1.06)
Hernia, stomach pain	1 (0.21)
Head reeling, weakness	8 (1.68)
Headache	1 (0.21)
Heart diseases	4 (0.85)
Hypertension	111 (23.31)
Hypertension, stomach problem	18 (3.82)
Prostate	42 (8.94)
Skin problem, weakness	1 (0.21)
Stomach pain	3 (0.63)
Swelling over the nose, body pain, weakness	1 (0.21)
Thyroid	1 (0.21)
Tuberculosis	1 (0.21)

The association between different habits and family history of diseases with morbidity status is shown in Table 4. After analyzing all study variables, four of the study variables, i.e., smoking (odds ratio (OR) = 1.229, 95% CI = 0.41-3.65), smokeless tobacco (OR = 0.815, 95% CI = 0.505-1.316), alcohol consumption (OR = 0.976, 95% CI = 0.485-1.967), and family history of hypertension (OR = 1.651, 95% CI = 0.927-2.938) were not significantly associated with morbidity status (p < 0.05). However, a family history of diabetes (OR = 2.466, 95% CI = 2.25-4.966) showed a significant association with morbidity (p = 0.0142).

**Table 4:** Association between risk factors and comorbidities. *: Statistically significant at p < 0.05.

Risk factors	Morbidity status, n (%)			χ^2^ (p-value)	Odds ratio	95% CI
		Single chronic disease	Multimorbidity			
Smoking	Yes	23 (4.89%)	4 (0.85%)	0.0121 (0.912)	1.229	0.413–3.654
	No	365 (77.66%)	78 (16.59%)			
Smokeless tobacco	Yes	165 (35.11%)	39 (8.29%)	0.508 (0.475)	0.815	0.505–1.316
	No	223 (47.45%)	43 (9.15%)			
Alcohol	Yes	51 (10.85%)	11 (2.34%)	0.004 (0.9476)	0.9768	0.485–1.967
	No	337 (71.70%)	71 (15.11%)			
Family history of diabetes	Yes	99 (21.06%)	10 (2.12%)	6.016 (0.0142)*	2.466	1.225–4.966
	No	289 (61.48%)	72 (15.32%)			
Family history of hypertension	Yes	117 (24.89%)	17 (3.61%)	2.505 (0.1135)	1.651	0.927–2.938
	No	271 (57.65%)	65 (13.82%)			

The hospitalized cases of three diseases among male and female participants found that the percentage of male participants with three diseases was higher than that of female participants (Table 5).

**Table 5:** Hospitalized cases in three different diseases.

Gender	Diabetes	Hospitalized	Hypertension	Hospitalized	Chronic kidney disease	Hospitalized
Male	110 (30.81%)	16 (4.48%)	90 (25.21%)	10 (2.80%)	41 (28.01%)	6 (1.68%)
Female	7 (6.19%)	7 (6.19%)	21 (18.58%)	3 (2.65%)	13 (11.50%)	1 (0.88%)

The association among age group and hospitalized cases of either sex showed that the >85-year age group of both males (9, 81.81%) and females (3, 27.27%) were hospitalized more, and there was no association among these two groups (χ^2^ = 3.902, p = 0.1422) (Table 6).

**Table 6:** Comparison of hospitalized cases between male and female patients.

Age (years)	Total	Percentage of patients hospitalized		χ^2 ^(p-value)
		Male (n = 32)	Female (n = 11)	
60–75	405	15 (3.7%)	2 (0.49%)	3.902 (0.1422)
76–85	54	8 (14.81%)	6 (11.11%)	
>85	11	9 (81.81%)	3 (27.27%)	

## Discussion

This study is the first effort at VIMSAR, Burla to provide a thorough assessment of the healthcare and social costs associated with multimorbidity in individuals ≥60 years of age. The prevalence of multimorbidity in this study likely results from variations in socioeconomic level, age, gender, and family structure. According to Bhojani et al. (2013), in South India, the prevalence of morbidity affects one-third of the population [15]. Conversely, Pati et al. (2021) in Odisha reported that the prevalence of multimorbidity was less than one-third in the case of females and one-fourth in males [16]. Pati et al. (2014) reported that 32% of respondents exhibited multimorbidity, with 30.6% classified as multimorbid, and 21.3% of these individuals were aged 60-69 years [17].

Our study also identified a substantial correlation between single morbidity and multimorbidity with age, gender, and family type; however, in our study, males were more susceptible to multimorbidity than females. The age group of 60-75 years was suffering more from single chronic diseases at 84.44%, and 90.9% of those in the age group >80 years were suffering from multimorbidity. This result was not similar to Pati et al. as 31.6% of those aged 60-70 years suffered from single chronic diseases, and 30.8% of those aged >70 years suffered from multimorbidity diseases in six states of India [17]. A study by Himanshu and Talukdar (2017) reported that the prevalence of multimorbidity in India was higher in the age group of >80 years (33.97%) compared to the age groups of 60-69 (19.82%) and 70-79 (28.17%) [18]. Population size and distribution among age groups was one of the factors contributing to the contrasting findings. A study by Verma and Mishra (2019) examined North Indian older adults and found that 33% had a single chronic disease, 31.8% had dyads, 15.5% had triads, and 5.8% had four or more chronic diseases [19]. However, our study contrasted with the findings, showing that the percentage of single diseases was higher in the age group of 60-75 (99.3%) compared to dyads in the 76-85-year age group (51.9%), triads in the 76-85-year age group (27.8%), and those with more than four diseases (54.5%). Furthermore, it has been recorded that multimorbidity becomes increasingly prevalent with advancing age [20-23]. We found that multimorbidity was significantly prevalent among individuals in the low-income group and those living in single-family households. In contrast, the prevalence of multimorbidity was decreased among older persons engaging in moderate or intense physical exercise relative to those maintaining a sedentary lifestyle.

Prior research from India revealed that common morbidities include diabetes, chronic lung ailments, arthritis, and hypertension [24]. In rural Tamil Nadu, the incidence of comorbidities among older adults included hypertension at 14%, heart disease at 9%, diabetes at 8.1%, and asthma at 6%. A similar finding was also found in our study. The highest prevalence of comorbidity was diabetes, followed by hypertension, CKDs, prostate diseases, arthritis, chronic lung disease, chronic heart disease, eye problems, cancer, weakness and swelling over the nose, acid peptic diseases, and thyroid disorders. The reasons behind the higher prevalence of these chronic diseases were socioeconomic status, family type, family responsibility, and stress.

In our study, behavioral factors such as tobacco use (including both smoking and smokeless forms) and alcohol consumption did not demonstrate a significant relationship with multimorbidity. Research indicates that individuals using tobacco and alcohol exhibit a 1.2-fold and 1.5-fold increased likelihood of developing multimorbidity compared to non-users [7,24]. A family history of diabetes showed a significant association with morbidity, which was similar to the study conducted by Daniyala et al. [25].

Study limitations

The primary limitation of this study was the duration of the study period. Due to the cross-sectional study design, causation could not be established. Additional large-scale studies with extended study duration are needed to validate the findings of our research. Additional research is necessary to enhance understanding of the epidemiology of multimorbidity and its effects on healthcare utilization and costs in western Odisha.

Study strengths

The strengths of the study were the sampling method, namely, systematic, representative, and population-based, as well as the use of well-recognized methodologies, trained interviewers, and sample size.

## Conclusions

Attention and action from policymakers are urgently needed to address multimorbidity in older persons. Improving public care facilities and increasing funding for the public health sector are two steps that the government can take to promote treatment for older people. In addition, healthcare providers may be better equipped to deal with problems among the elderly if they are educated on how to apply certain policies and guidelines. Improving the quality of life and reducing the burden of multimorbidity in disadvantaged older adults in rural and urban India may be achieved by addressing socioeconomic insufficiency, lifestyle hazards, and prompt illness treatment.
